# Health-promoting lifestyle and quality of life among Chinese nursing students

**DOI:** 10.1017/S1463423618000208

**Published:** 2018-04-06

**Authors:** Yim Wah Mak, Angela H. F. Kao, Lucia W. Y. Tam, Virginia W. C. Tse, Don T. H. Tse, Doris Y. P. Leung

**Affiliations:** 1 Associate Professor, School of Nursing, The Hong Kong Polytechnic University, Hung Hom, Hong Kong SAR, China; 2 Registered Nurse, Tseng Kwan O Hospital, Hospital Authority, Hong Kong SAR, China; 3 Registered Nurse, Haven of Hope Hospital, Hospital Authority, Hong Kong SAR, China; 4 Registered Nurse, Hong Kong Sanatorium & Hospital, Hong Kong SAR, China; 5 Assistant Professor, School of Nursing, The Chinese University of Hong Kong, Shatin, Hong Kong SAR, China

**Keywords:** health-promoting lifestyle, health-risk behavior, nursing students, quality of life

## Abstract

**Aim:**

This study aimed to examine the relationships between socio-economic status, health-promoting lifestyles, and quality of life among Chinese nursing students.

**Background:**

Nursing students will be future health promoters, but they may not always adopt the recommended healthy lifestyle. Currently, there are insufficient studies examining the health-promoting lifestyles of Chinese nursing students, and the impact of socio-economic status and health-promoting lifestyle on their health.

**Methods:**

This was a cross-sectional survey. Data were collected from nursing students studying in pre-registration nursing programs of a university in Hong Kong. The survey was conducted through a self-administered questionnaire that solicited information regarding their socio-economic status, health-promoting lifestyle, quality of life, and perceptions of the barriers to adopting a health-promoting lifestyle.

**Findings:**

A total of 538 students returned completed questionnaires for analysis. Among the health-promoting lifestyle subscales, the participants performed best in interpersonal relations and worst in physical activity, and the vast majority of them did not actively engage in health-risk behaviors. Hierarchical regression analyses revealed that only 5% of the variance in quality of life was explained by socio-economic variables, whereas a total of 24% of the variance was explained when health-promoting lifestyle variables were added. In particular, health responsibility, physical activity, spiritual growth, and stress management were statistically significant predictors of quality of life.

**Conclusions:**

Early concerns about how prepared nurses are to take on the role of promoting health still apply today. School administrators should plan the nursing curriculum to include activities that encourage student nurses to participate in health-promoting lifestyles. Future studies are needed to explore the barriers that prevent students from practicing health-promoting behavior.

## Introduction

Lifestyle refers to the ways in which individuals live that could affect their health. Health-promoting lifestyles (HPLs) refer to actions that individuals take the initiative to pursue that could benefit their health (Pender *et al*., 2006). The six components of health-promoting behaviors include health responsibility, physical activity, nutrition, interpersonal relations, spiritual growth, and stress management. The evidence indicates that people make an effort to pursue a healthy lifestyle achieve better health (Cockerham, 2005). In particular, four of the most prominent non-communicable diseases, namely, cardiovascular disease, cancer, chronic obstructive pulmonary disease, and diabetes, are linked to common preventable risk factors related to lifestyle including tobacco use, alcohol abuse, an unhealthy diet, and physical inactivity, which in turn have economic, social, gender, political, behavioral, and environmental determinants [World Health Organization (WHO), 2013]. Tackling the continuous growth in the global burden of non-communicable diseases constitutes one of the major challenges of the 21st century. While health and lifestyle are closely related, one of the major purposes of the WHO’s 2013–2020 Action Plan for the Global Strategy for the Prevention and Control of Non-communicable diseases is to strengthen the capacity of individuals and populations to make healthier choices and follow lifestyle patterns that foster good health (WHO, 2013).

Health professionals, especially in primary health care, have an important role to play in nurturing and enabling health promotion, and should work toward developing their special contributions in education and health advocacy (WHO, 2009). Today’s nursing students will become future health care providers and will take on roles as health promoters. However, there are still some concerns about how prepared nurses are for their role in health promotion (Mooney *et al*., 2011). According to the existing literature, the personal health practices of health professionals can affect their effectiveness and shape the interventions that they provide to their clients on health-related matters (Alpar *et al*., 2008). Since most lifestyle habits, including those of nursing students, are difficult to change because they are acquired early and can be followed for years, it is imperative that healthy behavior is promoted at the beginning stages of a student’s nursing education (Hui, 2002). However, little is known about the health-related lifestyles of future nurses in Chinese societies (Hui, 2002; Hsiao *et al*., 2010), and there is no information about the impacts of these practices on their health. Accordingly, this study was designed to identify the patterns of a HPL among Chinese nursing students and to examine the influences of socio-economic status and HPL on quality of life (QOL).

## Background

An HPL has been defined as a multidimensional pattern of self-initiated actions and perceptions that serve to maintain or enhance the level of wellness, self-actualization and fulfillment of the individual (Pender *et al*., 2006). In the last few decades, it has been emphasized that an HPL is a major strategy for improving health and reducing the incidences of illness that have become the greatest threat to the public’s health (Pender *et al*., 2006; Langford *et al*., 2011).

### HPL of nursing students

Most studies conducted in western countries on the HPL of nursing students have produced different or even contradictory results. Although nursing students are generally expected to be models of good health-related behavior, most of the studies that were reviewed indicated that they do not always demonstrate such behavior (Staib *et al*., 2006; Al‐Kandari and Vidal, 2007). Significant numbers of nursing students in various countries consumed alcohol and cigarettes, adopted irregular eating and sleeping patterns, did not achieve the recommended level of exercise, suffered from high levels of stress, and failed to seek appropriate preventive and curative clinical care for themselves (Staib *et al*., 2006; El Ansari *et al*., 2011).

However, several studies showed contradictory results, indicating that nursing students had healthier or at least comparable habits to those of non-nursing students (Shriver and Scott-Stiles, 2000; Can *et al*., 2008). Those results can mainly be attributed to socio-economic status, with age, gender, nationality, years of study, marital status, and family income, in particular, having been found to correlate closely with the HPL profiles of nursing students (Al‐Kandari and Vidal, 2007; Can *et al*., 2008; Wei *et al*., 2012). There is increasing evidence that socio-economic status is linked to health and health-related behavior (Cohen *et al*., 2010; El Ansari *et al*., 2011). However, to our knowledge, no previous studies have been conducted on the degree of the socio-economic status of nursing students and the impact of an HPL on their health. Other factors contributing to the contradictory results may be methodological, including sample size, the timing of the collecting of data, and measurement issues related to the instruments that were used in the study.

### HPL and QOL

QOL refers to a person’s overall sense of well-being, including all aspects contributing to their subjective satisfaction, such as physical health, psychological state, social relationships, and relationships to salient features (WHO, 2004). An HPL contributes to a positive QOL because the individual who engages in an HPL will remain healthy and functional without the burden of disease and disability (Eriksson *et al*., 2010; Conry *et al*., 2011).

Studies have been conducted on the effects of an HPL on the QOL of undergraduate students, but no study has specifically focused on nursing students. Domains contributing to university students’ satisfaction with life included feeling in control of life events, feeling satisfied with school, being perceived as healthy, and recognizing a sense of social belonging (Keith and Schalock, 1994). Psychological factors, such as a sense of coherence and level of optimism and self-efficacy in physical and social functioning, were also related to QOL in university students (Posadzki *et al*., 2009).

Since beliefs about health, illness, and lifestyle are largely influenced by culture and local value systems (Singer, 2012), it is difficult to assess the extent to which results from western studies can be extrapolated to the Chinese population. No previous study has yet addressed the effects, in particular, of socio-economic status and an HPL on QOL among nursing students. It is essential to identify specific health promotion needs in order to develop interventions to enable young nursing students to achieve a healthier lifestyle during their training in early adulthood and in the future.

## METHOD

### Design

This was a cross-sectional survey. Data were collected by a self-administered questionnaire. Participants were recruited by convenience sampling.

### Participants

There are three universities in Hong Kong that provide pre-registration nursing programs. Participants were recruited from four pre-registration nursing programs from one of the three universities. A total of 813 students were invited to take part in the survey. In total, 556 questionnaires were collected. However, 18 of them were discarded due to incomplete answers; thus, only 538 questionnaires were analyzed, representing a 66% response rate.

### Instruments

The questionnaire used in this study consisted of closed-ended and structured questions written in English, soliciting information about (1) HPL, (2) health-risk behavior, (3) QOL, and (4) socio-economic status.

#### HPL

The Health-Promoting Lifestyle Profile II (HPLP-II) developed by Walker *et al*. (1987) was adopted. It measures how frequently respondents engage in 52 aspects of an HPL from the domains of health responsibility, exercise, nutrition, spiritual growth, interpersonal relations, and stress management. Each item was measured using a four-point response format, with higher scores indicating more frequent performance of that aspect of the HPL. This instrument was chosen because it has been used extensively in health-promotion research and contains measures of all six of the core components of health-promoting behaviors. The content validity was established by a literature review and by the evaluation of content experts, and the *α* coefficient for internal consistency reached about 0.8 for the individual subscales and 0.9 for the total instrument (Shirlee and Neva, 2004). The three-week test-retest reliability coefficient for the total scale was 0.89 (Stuifbergen *et al*., 2003).

#### Health-risk behavior

Five questions about health-risk behavior were modified from the Youth Risk Behavior Survey (YRBS) of the Youth Risk Behavior Surveillance System, which was developed by the Centers for Disease Control and Prevention in 1989 (Kolbe *et al*., 1993). The YRBS questionnaire has been found to be valid and reliable for use among Chinese youth (Lee *et al*., 2001). The five questions included how often the respondents smoked, how often they used illegal drugs, how often they practiced safe sex, and how often they used unhealthy/unsafe ways to lose weight. Each item was measured using a five-point response format, with higher scores indicating more frequent performance of the above behavior.

#### QOL

QOL was measured by the World Health Organization Quality of Life (WHOQOL)-BREF instrument (WHO, 2004). It consists of 26 items that measure four domains of QOL: physical health, psychological health, social relationships, and environment. The response format is a five-point Likert scale, where higher points indicate higher QOL. This instrument was chosen because it has been proven to be applicable to people living under different circumstances, conditions, and cultures (Saxena *et al*., 2001). It contains social relationship and environmental domains that rarely appear in other multidimensional health instruments. Its content validity ranged from 0.53 to 0.78 for item-domain correlations and from 0.51 to 0.64 for inter-domain correlations (Yao *et al*., 2002). In addition, the *α* coefficient for internal consistency ranged from 0.66 to 0.84 for the four domains, and from 0.86 to 0.91 for the total score (The WHOQOL Group, 1998; Amir *et al*., 2000). The two-week test-retest reliability for domains and individual items ranged from 0.71 to 0.88 and from 0.56 to 0.84, respectively (The WHOQOL Group, 1998; Amir *et al*., 2000).

#### Socio-economic status

The socio-economic data that were collected included information on a respondent’s gender, age, marital status, monthly family income, highest level of education of the respondent’s father and mother, the nursing program that the respondent is studying in, the year of study, and total number of hours worked weekly for paid jobs, if any.

### Validation of the questionnaire

Prior to the survey, the questionnaire was conducted with 42 university students who were not eligible to take part in the present study. The inter-class correlation for HPLP-II was found to be 0.905 for the whole scale and from 0.716 to 0.827 for its subscales. The inter-class correlation for the WHOQOL-BREF was found to be 0.870 for the whole scale and from 0.598 to 0.782 for its domains.

### Data collection

Data collection was carried out on two occasions in April to August 2011. For the final year students, data collection was carried out after a career talks to those students. The research group was present at the venue to distribute the information sheets and questionnaires to the students. For the non-final year students, the data were collected during the students’ clinical placements. The clinical mentors were invited to distribute the information sheets and the questionnaires to their students.

### Data analysis

Data analysis was conducted using SPSS version 23.0. Descriptive statistics were used for the socio-economic variables and the data on health-risk behavior, HPL, and QOL. A hierarchical linear regression analysis (HLM) was conducted to identify factors associated with QOL among nursing students (Raudenbush and Bryk, 2002). In order to assess the additional contribution of HPL in predicting QOL, the socio-economic variables were entered at Step 1 and the six HPL subscales at Step 2.

### Ethical considerations

The study was approved by the University’s research ethics committee. The ethical considerations for the study included implied consent, autonomy, anonymity, and confidentiality. The participants could refuse to participate or could withdraw at any time during the study. Consent to participate was assumed if the participants completed and returned the questionnaire. Neither the participants’ names nor information that could identify them were required on the questionnaires. Before the students started to complete the survey questionnaires, either a member of the research team or a teacher of their class gave them verbal instructions together with an information sheet regarding the objectives of this study and their involvement. Explanations of this research included information regarding their anonymity, voluntary participation, and the harmlessness of joining the study. Upon completing the questionnaires, the students placed them directly in a collection box held by the researchers or clinical mentors. The students could also return the completed questionnaires to an assigned collection box in the general office at the nursing department of the participating university. Only the members of our research team could access the collected questionnaires.

## Findings

### Characteristics of the participants

The background characteristics of the participants are shown in Table 1. Of the participants, 73% were females and 27% were males. Their ages ranged from 18 to 31 years, with a mean age of 21.7 years (SD=2.08). The sample was composed of participants from different years of study. Almost all of them were single (99%), with more than half (63%) coming from families with a monthly income of less than HK$20 000 (US$2563.36).

**Table 1:** Participants’ socio-economic characteristics (*N*=538)

Characteristics	*n* (%)
Gender	
Female	392 (72.9)
Male	146 (27.1)
Age group	
Under 20	171 (31.8)
21–25	345 (64.1)
Over 25	22 (4.1)
Year of study	
1	295 (54.8)
2	74 (13.8)
3	121 (22.5)
4	48 (8.9)
Marital status	
Single	531 (98.7)
Married	5 (0.9)
Other	2 (0.4)
Monthly family income^a^	
Under HK$10 000	146 (27.1)
HK$10 000–19 999	194 (36.1)
HK$20 000–29 999	106 (19.7)
HK$30 000–39 999	45 (8.4)
HK$40 000 and above	57 (8.7)
Highest education level of father	
Primary or below	221(41.0)
Secondary	271 (50.4)
Post-secondary	45 (8.4)
Highest education level of mother	
Primary or below	232 (43.1)
Secondary	275 (51.1)
Post-secondary	31 (5.8)

a US$1=HK$7.8.

Table 2 presents the participants’ engagement in health-risk behavior. Almost all of them did not smoke (95%) or use illegal drugs (99%). The majority of them did not consume alcohol regularly (77%). Most of them (79%) reported that they had never had sexual intercourse. Of those who had, almost all (93%) reported that they had used protective measures during sexual intercourse. Almost half of them (46%) reported that they had attempted to lose weight. Of those who had attempted to lose weight, 40% had tried to use inappropriate methods such as dieting without guidance from a nutritionist, taking pills/laxatives without a prescription, or induced vomiting.

**Table 2:** Participants’ health-risk behaviors (*N*=538)

Health-risk behaviors	*n* (%)
Cigarette use	
Never	513 (95.4)
Past but not current	12 (2.2)
Current	13 (2.4)
Alcohol use	
Never	305 (56.7)
Past but not current	111 (20.6)
Current	122 (22.7)
Illegal drug use	
Never	530 (98.5)
Past but not current	4 (0.75)
Current	4 (0.75)
Using preventive measures during sexual intercourse	
Never had sexual intercourse	426 (79.2)
Currently using protective measures	104 (19.3)
Never used preventive measures	8 (1.5)
Losing weight using unhealthy/unsafe diet methods	
Never attempted to lose weight	293 (54.5)
Never used these methods	148 (27.5)
Currently using these methods	97 (18.0)

Table 3 presents the participants’ HPL and QOL. The mean for the total HPL was 128.2, with an SD of 17.4 (possible range 52–208). The performance of individual participants differed greatly, ranging from 83.0 to 182.0. In the HPL subscales, the participants scored the highest in interpersonal relations (mean=2.8) and the lowest in physical activities (mean=2.1), with ascending scores for health responsibility, stress management, nutrition, and spiritual growth falling in between. With respect to the mean scores of the QOL domains (possible range 4–20) the social domain ranked highest (mean=13.7), followed by the environmental (mean=13.5), psychological (mean=13.1), and physical domains (mean=12.2).

**Table 3:** Participants’ health-promoting lifestyle and quality of life (*N*=538)

Variables	Mean (SD)
Health-promoting lifestyle^a^	
Interpersonal relations	2.78 (0.44)
Spiritual growth	2.69 (0.45)
Nutrition	2.50 (0.42)
Stress management	2.41 (0.43)
Health responsibility	2.29 (0.44)
Physical activity	2.06 (0.51)
Total score	128.23 (17.37)
Quality of life^b^	
Social relationships	13.74 (2.30)
Environmental	13.52 (2.04)
Psychological health	13.10 (1.76)
Physical health	12.15 (1.86)

a Possible mean scores of subscales ranged from 1 to 4 and possible mean score of the total scale ranged from 52 to 208, with higher score indicating higher frequency of health-promoting lifestyle.

b Possible mean scores of subscales ranged from 4 to 20, with higher score indicating better quality of life.

### Predictors of QOL

A HLM was used to predict QOL among the participants. We used linear regression to identify predictors of QOL instead of a data analysis method involving dividing the participants into two groups (good or bad QOL). This is because a binary split of the participants into two groups (good versus bad QOL), for example, at the median value of QOL, could not explain an underlying dichotomy in QOL. Furthermore, there is no recognized cut-off point between a good and bad QOL. Thus, using any arbitrary cut-off point may lead to a mis-estimation of the results of the model (Royston *et al*., 2006). Thus, instead of taking a dichotomous approach to dealing with the continuous variables, we use linear regression, where the outcome variable can be kept continuous so as to more clearly determine the relationship between the outcome and predictor variables. In model 1, the socio-economic variables were found to explain only 5% of the variance in QOL, where non-adjusted and adjusted *R* ^2^ were 0.050 and 0.038, respectively. Only family income (estimated coefficient=0.092, SE=0.024, *P*<0.001) emerged as a significant predictor (Table 4).

**Table 4:** Predicting quality of life: hierarchical linear regression (*N*=538)

	Step 1			Step 2		
Predictors	Estimate	SE	*P*-value	Estimate	SE	*P*-value
Socio-economic variables						
Gender	−0.096	0.068	0.158	−0.116	0.067	0.085
Age	−0.029	0.016	0.067	−0.015	0.014	0.287
Years of study	0.003	0.032	0.934	0.037	0.030	0.217
Working hours per week	−0.030	0.020	0.130	−0.033	0.018	0.064
Income	0.092*	0.024*	<0.0001*	0.068*	0.022*	0.002*
Father’s education	−0.014	0.050	0.783	−0.032	0.045	0.475
Mother’s education	0.043	0.052	0.409	0.015	0.047	0.742
Health-promoting lifestyle variables						
Health responsibility				−0.265*	0.083*	0.002*
Physical activity				0.169*	0.071*	0.018*
Nutrition				−0.096	0.077	0.212
Spiritual growth				0.428*	0.097*	<0.0001*
Interpersonal relations				0.176	0.096	0.066
Stress management				0.277*	0.092*	0.003*
*R*		0.224			0.489	
*R* ^2^		0.050			0.239	
Adjusted *R* ^2^		0.038			0.220	
*F*-value(df1, df2)	3.993(7, 529)			12.663(13, 523)		

* *p*<.05, indicates a significant predictor of quality of life.

The addition of the six HPL variables (in model 2) led to a significant increase to about 24% of the total variance explained, and the non-adjusted and adjusted *R* ^2^ were 0.239 and 0.220, respectively. Significant associations were observed between QOL and four out of the six HPL variables after controlling for the socio-economic variables: health responsibility (estimate coefficient=−0.265, SE=0.083, *P*=0.002), physical activity (estimate coefficient=0.169, SE=0.071, *P*=0.018), spiritual growth (estimate coefficient=0.428, SE=0.097, *P*<0.0001), and stress management (estimate coefficient=0.277, SE=0.092, *P*=0.003). Family income remained a significant predictor of QOL after the addition of the six HPL variables.

## Discussion

This study identified the patterns of HPL, health-risk behavior, and QOL among a group of Chinese nursing students and provided evidence of the effects of socio-economic status and HPL on QOL.

### Social and physical health

The overall HPL performance of the participants in our study was similar to that of the undergraduate nursing students who participated in Hui’s (2002) study in Hong Kong (120.0 versus 116.3). Our study participants performed best in the interpersonal relations dimension and worst in the physical activity dimension – also consistent with the findings of Hui’s (2002) study. The findings from the QOL measurement aspect of this study also revealed that our participants performed best in the social relationships domain and worst in the physical health domain. Furthermore, 18% of the participants were currently using unhealthy or unsafe methods to lose weight. Similar to the situation in other developed countries, unhealthy weight control behaviors (UWCB) were found to be common among adolescents, particularly girls. A population-based study found that 33.9% of Spanish and 45.7% US girls; 14.2% Spanish, and 31.3% US boys used dieting to control their body weight (López‐Guimerà *et al*., 2013). This high prevalence in the use of UWCB is a cause of a great concern since studies have found that dieting during adolescence leads to persistent dieting and disordered eating into adulthood (Story *et al*., 2014). UWCB may also lead to negative consequences for both physical and psychological health (French and Jeffery, 1994; Rawana *et al*., 2010).

The results of this study indicate that there is a need to add a nutrition education component to the nursing curriculum to help nursing students help themselves as well as educate patients and clients.

As good interpersonal relations are a prerequisite for effectively teaching others about health, nursing students equipped with this skill will be in a better position to help the public to develop positive health attitudes and well-being (Lane, 2010). Students are advised to continue to enhance their interpersonal skills through observations and daily practice, such as by volunteering in health-promoting activities and joining a students’ club in school.

A lack of physical exercise has been commonly reported among nursing students and other populations of young people all over the world (Lee and Wang, 2005; Staib *et al*., 2006; Rey-Lopez *et al.*, 2010). This may reflect a global trend in which young people do not prioritize physical activity in their lifestyle (Carnethon *et al*., 2010). Nursing students are physically inactive possibly because they are likely to be preoccupied with their stressful nursing training, which takes up much of their time and energy and prevents them from participating in regular exercise programs. Since action learning has been advocated as an effective way for nurses to address what is sometimes perceived as insuperable barriers to developing health-promoting opportunities (Carlson and Warne, 2007), a review of the curriculum may be needed to integrate lunchtime or end-of-day exercises, or fitness classes in the context of school, to promote regular exercise and enhance the physical health of nursing students.

### Health-risk behavior

The percentage of the participants in this study who engaged in health-risk behavior was less than the percentage previously reported in the general population in Hong Kong. About 98 and 77% of the students in the current study were not currently smoking or drinking, respectively, compared to 89.6 and 29.5% of the general population (Centre for Health Protection, 2017). In addition, only 0.75% of the students in this study had previous experience in the use of illegal drugs, while in the general population it has been reported that 9.3% of young people aged between 18 and 24 have taken illegal drugs (Lau *et al.*, 2005). In contrast, a study conducted in the United States found that nursing students had less healthy behavior in the areas of exercise, weight, and smoking when compared with the general population (Staib *et al*., 2006).

Nursing students were expected to be more concerned about their health than the general public, as they are exposed to more health-promotion knowledge in their program. As there are well-established links between health-risk behavior and the incidence of a variety of disorders, such as circulatory and respiratory disorders, that are likely to imperil their health, it is important that nursing students be encouraged to continue to comply with upholding health-promoting behavior throughout their studies and in their future career (WHO, 2013). Nursing students who are less engaged in health-risk behavior are also in an excellent position to act as health exemplars in their future practice, as clients are more likely to comply with health-related behavior if such behavior is modeled by health professionals.

### Impact of HPLs on QOL

In line with earlier research, we found that an HPL does influence health (Mokdad *et al*., 2004). We also found that an HPL has greater health impacts than socio-economic factors on QOL. As our findings reveal, different dimensions of HPL such as health responsibility, exercise, spiritual growth, and stress management are significantly associated with the QOL of nursing students.

Studies have shown that unhealthy lifestyles such as smoking, a poor diet, a sedentary life, excessive alcohol consumption, and so forth are leading to morbidity and mortality (Mokdad *et al*., 2004), and could shorten an individual’s life by six years (Manuel *et al*., 2016). The unhealthy lifestyle of nursing students during late adolescence can have long-term adverse effects on their health in adulthood (Hancox *et al*., 2004). Earlier modifications could result in better health outcomes. The overall picture presented in the literature suggests that the call for the effective inclusion of health promotion in nursing education has in many cases gone unheeded (Whitehead, 2007). Therefore, health-promotion programs aimed at striking a balance between academic performance and total well-being should be targeted at nursing students for the sake of their own health as well as to prepare them to assume the role of health promoters in the future (Neinstein, 2008).

### Limitations of the study

This study has several limitations. First, the cross-sectional design means that the data were examined at one point in time and on only one occasion; such a design does not allow for observations to be made of changes in the HPL and QOL of nursing students throughout their training. It precludes the making of any conclusive causal linkages between HPL and QOL. Second, the survey design tends to produce superficial rather than in-depth information on a particular phenomenon, and therefore suits an extensive rather than intensive analysis. Third, a self-reporting bias may have occurred in the self-administered questionnaire. The respondents may have had the underlying desire to report that they had a healthy lifestyle; thus, it is possible that what the students considered to be acceptable health behavior could have been overrepresented in the results. Finally, the response rate of 66% suggests that bias may possibly have been introduced, in that nursing students who failed to respond may have encountered more barriers to practicing HPL than those who did respond. However, a response rate of around 66% is not unusual for surveys of university students (Patkar *et al*., 2003).

### Recommendations for school administrators

School administrators are needed to explore barriers that could prevent students from practicing HPLs. Longitudinal studies may also be needed to further explicate the causal linkages among variables, to predict the long-term effects of HPLs on QOL during nursing students’ university years and to promote the health of future nurses in the long term. A systematic review found that interventions are available that can effectively enhance HPLs among university students (Plotnikoff *et al*., 2015). Given that universities are settings with accessible facilities where a large number of adolescents can be reached at a key time to develop healthy lifestyle behaviors, relevant effective interventions should be considered for implementation at universities.

### Conclusion

The current study contributes to the expansion of a coherent body of knowledge about HPLs and QOL among nursing students. Health responsibility, spiritual growth, stress management, and physical activity in particular have been found to be helpful in enhancing their QOL. Through the early identification of the HPL, health-risk behavior, and health of nursing students, school administrators would be better able to contribute to the development and implementation of tailor-made programs that best meet the needs of these students. School administrators should plan the curriculum for nursing students by including activities that can cultivate their participation in a HPL.
